# Minocycline as an adjunct for treatment-resistant depressive symptoms: study protocol for a pilot randomised controlled trial

**DOI:** 10.1186/s13063-015-0933-5

**Published:** 2015-09-15

**Authors:** Muhammad I. Husain, Imran B. Chaudhry, Raza R. Rahman, Munir M. Hamirani, Inti Qurashi, Ameer B. Khoso, John FW Deakin, Nusrat Husain, Allan H. Young

**Affiliations:** Centre for Affective Disorders, Institute of Psychiatry, Psychology and Neuroscience, King’s College London, De Crespigny Park, London, SE5 8AF UK; The Mount, Whalley Road, Accrington, Lanacashire BB5 5DE UK; Dow Institute of Health Sciences, Karachi, Pakistan; Department of Psychiatry, Abbasi Shaheed Hospital, Karachi, Pakistan; Ashworth Research Centre, Mersey Care NHS Trust, Parkbourn, Maghull, L51 1HW UK; Pakistan Institute of Learning and Living, Karachi, Pakistan; University of Manchester, Oxford Road, Manchester, UK

**Keywords:** Depression, Major depressive disorder, Minocycline, Anti-inflammatory

## Abstract

**Background:**

Depression is one of the leading causes of disability worldwide. A high proportion of patients do not respond to standard drug treatments. Recent evidence has suggested that anti-inflammatory treatment may have beneficial effects in major depression. Minocycline is a tetracycline antibiotic with good CNS penetration that exerts effects on multiple interacting symptoms implicated in the pathophysiology of mood disorders. Open-label studies have suggested that minocycline is effective as an adjunct drug in improving depressive symptoms.

**Methods/Design:**

This is a multi-centre, 3-month, double-blind, placebo-controlled, pilot trial of minocycline added to treatment as usual for patients suffering from DSM-IV major depressive disorder. This will be a double-blind, randomised, controlled, two parallel-arm study with 20 participants in each arm, giving a total of 40 participants. There will be a screening visit, a randomization visit and four follow-up visits. Clinical assessments using the Hamilton Depression Rating Scale (HAM-D), Clinical Global Impression scale (CGI), Patient Health Questionnaire-9 (PHQ −9) and the Generalised Anxiety Disorder scale (GAD-7) will be carried out at every visit. Side effects checklists will also be undertaken at each visit. Biomarkers (inflammatory cytokines and CRP) will be measured at baseline and at the end of the treatment phase. Minocycline will be started at 100 mg once daily (OD) and will be increased to 200 mg at two weeks.

**Discussion:**

Anti-inflammatory treatments have been shown to have some beneficial effects in the treatment of major depressive disorder. The aim of this pilot randomised controlled trial is to establish the degree of improvement in depressive symptoms with the addition of minocycline to treatment as usual.

**Trial registration:**

ClinicalTrials.gov NCT02263872 registered 10 October 2014.

## Background

Major depressive disorder is associated with significant morbidity and mortality. Depression is the leading cause of disability worldwide in terms of years lost due to disability [1]. Although depressive symptoms are amenable to antidepressant treatment, a high proportion of patients neither responds adequately nor achieves remission. For example in the Sequenced Treatment Alternatives for the Relief of Depression (STAR*D) study, the response and remission rates with stage 1 treatment (citalopram) were 49 and 37 %, respectively. The further response rates decreased to 16 and 13 %, respectively, over the subsequent next three treatment steps [2]. More recently, a systematic review of the efficacy of current pharmacological treatments of depressive disorder in primary care showed only a relatively small effect size for antidepressant treatments when compared with the placebo [3]. Thus, there remains a clear need for exploring novel treatment approaches.

Recently, there have been promising preclinical and clinical data linking inflammatory processes to a range of psychiatric illness including depression. The evidence that depression (or some subgroups thereof) is an inflammatory-related disorder comes from multiple sources, including the observation that depression is associated with raised inflammatory markers in the absence of a medical illness [4]. More specifically, depression has been associated with higher levels of positive acute phase proteins (APPs) and low levels of negative APPs [5], as well as increased levels of complement factors C3c and C4 and immunoglobulin M (IgM) and IgG [6]. Inflammatory medical illnesses, both CNS and peripheral, are associated with greater rates of depression and in patients with Crohn’s disease and comorbid depression, bouts of physical disease activity tend to co-occur with depressive episodes [7]. Furthermore, patients treated with cytokines for various illnesses have an increased risk of developing depressive illness [8]. For example, treatment with cytokine IFN-α corresponded with the development of depressive symptoms in up to 45 % of patients [9].

The available evidence suggests that the addition of an anti-inflammatory medication may be efficacious in the treatment of depressive illness. Muller et al. [10] demonstrated a reduction in depressive symptoms when using Celecoxib, a COX-2 selective non-steroidal anti-inflammatory drug, in addition to Reboxetine, for the treatment of major depressive disorder in a double-blind, randomised, placebo-controlled pilot study. A recent meta-analysis showed that augmentation with Celecoxib is an effective add-on treatment for unipolar depressive patients [11]. However, other studies have found that anti-inflammatories may have an antagonistic effect on the antidepressant actions of SSRIs [12]. Further work is needed in this area to clarify the role of inflammatory processes and anti-inflammatories in the treatment of depression.

The tetracycline antibiotic minocycline has been proposed for the treatment of depressive symptoms as well as negative symptoms in schizophrenia [13, 14]. Preliminary data from an open-label study of patients with psychotic unipolar depression suggested that minocycline augmentation of antidepressant treatment was effective and well tolerated [15]. Minocycline is a pleiotropic agent that exerts effects on multiple interacting systems (that is, anti-inflammatory, anti-oxidant, anti-apoptotic, glutamatergic and monaminergic) implicated in the pathophysiology of mood disorders. Despite such neuroprotective properties, there have been no published clinical trials to date investigating the antidepressant effects of minocycline in individuals with major depressive disorder.

In this double-blind, randomised, controlled pilot trial, we will determine the efficacy of minocycline as an adjunct to treatment as usual (TAU) in patients with major depressive disorder. We hypothesise that minocycline augmentation will lead to an improvement in depressive symptoms in participants in comparison with the placebo.

### Aim

The aim of this study is to investigate whether the addition of minocycline to treatment as usual (TAU) for 3 months in patients with major depressive disorder will lead to an improvement in depressive symptoms compared with TAU.

## Methods/Design

### Overview

This is a multi-centre, 12-week, double-blind, placebo-controlled, pilot trial of minocycline added to treatment as usual for patients suffering from DSM-IV major depressive disorder. It will be a double-blind, randomised, controlled, two parallel-arm study with 20 participants in each arm, giving a total of 40 participants. The study will be conducted in Karachi, Pakistan, and participants will be recruited from outpatient psychiatric clinics at the following units: the Karwan-e-Hayat Hospital, Abbasi Shaheed Hospital, Civil Hospital Karachi and Institute of Behavioural Science, Karachi.

All patients will give written informed consent after reading the information provided in Urdu. Treatment as usual (TAU) will comprise medications including antidepressants (SSRI’s, Tricyclics, MAOIs, and SNRIs), mood stabilisers (with the exception of valproic acid) and antipsychotics, as well as psychotherapy and other psychosocial interventions.

### Randomisation and masking

Tablets will be dispensed by a single pharmacy in Karachi according to separate computerised randomisation lists generated using an online randomisation tool (www.randomisation.com) and will be held by a central pharmacist in Pakistan. There will be no further stratification.

Patients, their families, referring psychiatrists and the research assistants carrying out the assessments will be blind to the study drug until the completion of the study. Reign Nutro Pharma and Jawed Traders (Pakistan) will provide minocycline and placebo in identical tablet form, matched both for colour and size.

### Sample size

Participants will be randomly divided into two groups: the intervention group and treatment-as-usual group. A total of forty participants will be recruited and divided equally into two arms. This is a pilot trial, and therefore, the sample size is sufficient for a study of this nature. Assuming an attrition rate of 10 %, we are confident that we will have at least 18 subjects per group for analysis at the end of the trial. The U.S. Food and Drug Administration guidance on drug study design recommends that a minimum of 12 subjects per group is sufficient for pilot trials [16].

### Power calculation

In the proposed study, a group size of 18 participants is powered at 0.75 and an alpha of 0.05 to detect an effect size of 0.75 in the reduction of Hamilton Depression scores (HAM-D) [17] between the minocycline and treatment as usual. At present, there are no clinical trials of minocycline in the treatment of unipolar or bipolar depression; therefore, a true population effect size is currently unknown. However in clinical trials with antidepressants, an effect size of 0.40 or higher is considered a clinically significant response criterion [18].

### Local research ethics committee approval

Institutional review board (IRB) approval has been obtained from the ethics committee of the Karachi Medical and Dental College and Dow University of Health Sciences, Pakistan.

### Inclusion criteria

Inclusion criteria are (1) patients aged 18 to 65 years, (2) Diagnostic and Statistical Manual-IV (DSM-IV) diagnosis of major depressive disorder, (3) competent and willing to give informed consent, (4) taking the current antidepressant medication for a minimum of 4 weeks (6 weeks for Fluoxetine) prior to baseline, (5) the current episode of depression has failed to remit with at least two courses of antidepressant treatment (one of which is the current course), (6) able to take oral medication and (7) if female, willing to use adequate contraceptive precautions and to have monthly pregnancy tests.

### Exclusion criteria

Exclusion criteria are (1) relevant medical illness (renal, hepatic, cardiac, serious dermatological disorders such as exfoliative dermatitis, systemic lupus erythematosis), (2) prior history of intolerance to any of the tetracyclines, (3) concomitant penicillin therapy, (4) concomitant anticoagulant therapy, (5) presence of a seizure disorder, (6) currently taking valproic acid, (7) any change of psychotropic medications within the previous 4 weeks, (8) diagnosis of substance abuse (except nicotine or caffeine) or dependence within the last 3 months according to DSM-IV criteria, (9) pregnant or breast-feeding or (10) presence of primary psychotic disorder.

The criteria for leaving the trial are (1) patient’s request, (2) at the discretion of the responsible medical officer or investigator (for example, an adverse event or poor compliance) and (3) pregnancy.

### Study procedure

#### Recruitment

The psychiatry consultants responsible for patient care will be approached and asked if they will allow their patients to take part in this research study.

In the first instance, the research clinician will approach the clinical teams to inform them about the research study, especially with regard to inclusion and exclusion criteria. The research clinician will establish good working relationships with the individual clinical teams. They will be in regular contact either by phone or by visits to determine, in collaboration with the clinical team, if the patients are suitable to take part in the research study. If patients meet the entry criteria, are clinically stable and the consultant psychiatrist and the clinical team agree the patient could be a possible participant, the consultant will introduce the study to the patient. With the patient’s agreement, the research clinician will visit the patient to explain the research study verbally and provide them with the patient information sheet. After the patient has had time to read and understand the patient information sheet (at least 24 h) and is willing to take part, a meeting (visit one) will be set up with the patient in order to obtain signed informed consent for the research and also signed consent for the research team to have access to their medical notes.

#### Screening visit

Confirmation of patient suitability will be carried out at this point. Participants recruited to the trial will undergo structured diagnostic interviews using the Mini International Neuropsychiatric Interview (MINI) to confirm a diagnosis of DSM-IV major depressive disorder [19]. This tool has been validated for use in the local Urdu language and has been used in previous studies [20]. The Hamilton Depression Scale (HAMD-17) [17] will be used to measure severity and response and is not being utilised as a screening tool for this study and therefore there is no specific HAMD-17 score required for study inclusion. Other Inclusion/Exclusion criteria will be checked at this visit and confirmation of consent and pregnancy testing, if appropriate will be carried out.

#### Follow-up

Participants will be randomised to receive minocycline or treatment as usual (TAU). Patients will continue with their current antidepressant treatment prescribed by their psychiatrists. Minocycline added to TAU will start at a dose of 100 mg daily and will be increased after two weeks to 200 mg daily, taken as a single dose to encourage compliance.

The patients’ day-to-day care will remain the responsibility of the consultant psychiatrist or other mental health professional in charge of the patient. However, research assistants will maintain contact throughout the study in order to respond to any concerns or changes in circumstances or mental or physical state. Contacts will be 2-weekly for the duration of the study. Any study-related safety concerns will be the responsibility of the co-investigators who can be contacted at any time through the research team.

#### Outcome measures

The primary clinical outcome measures will be mean change from baseline to week 12 on the Hamilton Depression Scale scores [17]. Response is defined as a reduction of 50 % or more of the HAMD-17. Remission is defined as a score of ≤7 of the HAMD-17.

Ratings will be made on the basis of a semi-structured clinical interview at baseline, weeks 2, 4, 8 and 12.

The secondary clinical outcome measures will be the Clinical Global Impression (CGI) scale, a 7-point overall measure of severity; the PHQ −9 [21], a depression severity measure; and the GAD-7 [22], a measure of generalised anxiety disorder. These instruments have been used in a previous clinical trial in Pakistan [23]. Participants will also complete the Mood Disorder Questionnaire (MDQ) [24], a brief self-report questionnaire to screen for bipolar disorder. Adverse effects will be monitored using a rating scale that has been specifically designed for minocycline. This rating scale has been used by the authors in previous studies [14].

#### Biomarkers

Participants will be asked to provide two blood samples for research analysis. The provision of blood samples is optional and does not affect participation in the trial. These samples will be collected at baseline and at week 12. The samples will be collected to investigate the relation of the minocycline to the inflammatory markers and if this relates to the subjective experience of symptom change. The biomarkers tested include complete blood count, erythrocyte sedimentation rate (ESR) and C-reactive protein (CRP).

#### Research assistant training and inter-rater reliability

Research assistants in Karachi were trained in Structured Clinical Interview for DSM-IV (SCID) and clinical assessments at the University of Manchester for a previous grant-funded study. Interrater reliability will be measured throughout the study by local PIs. To establish inter-rater reliability training videos will be used while two raters code them independently. SPSS will then be used to calculate the Intraclass Correlation Coefficient.

#### Statistical analysis

All analyses will be based, as far as possible, on the intention-to-treat principle (with weighting adjustments to allow for differential loss to follow-up). Primary and secondary outcomes will be measured using a mixed-effects model (ANCOVA).

#### Study coordination

The study will be coordinated through weekly meetings by local investigators. There will be two weekly tele- or video-conferences with the chief investigator.

#### Dissemination

Outcomes will be reported in a peer reviewed journal (publication target Journal of Psychopharmacology) and will also be communicated at relevant conferences and local newspapers for general audience.

## Discussion

Depression remains a leading cause of morbidity and mortality globally. Standard pharmacological treatments often have poor response leading to diminished social and functional outcomes. The consequential financial burdens of untreated or partially treated depressive symptoms on the individual and wider society are significant. Recent studies indicate that anti-inflammatory treatments may have some beneficial effects in depressive disorder. A previous open-label study and case reports indicate that the use of minocycline as an adjunct to antidepressants may provide some evidence of a reduction in depressive symptoms.

To date there are no controlled clinical trials that have investigated the use of minocycline for the treatment of depressive symptoms. Our pilot randomised controlled trial is the first of its nature and the findings from it may contribute to evidence in the management of patients with treatment resistant depressive symptoms. Should our results show a trend that minocycline reduces depressive symptoms, we will use them to inform the development of a larger trial. We hope that the current pilot study will determine the feasibility of recruitment, randomization, intervention implementation, blinded assessment procedures and retention for a larger scale hypothesis testing study. In the long term, we hope the findings of this study will contribute to the treatment of patients who suffer from prevalent and often debilitating illness.

### Trial status

This clinical trial was registered in October 2014. The study is currently recruiting participants. The estimated study completion date is August 2016. Please refer to this study by its ClinicalTrials.gov identifier: NCT02263872.

## Abbreviations

ANCOVA: analysis of covariance
CGI: Clinical Global Impression Scale
DSM-IV TR: Diagnostic and Statistical Manual-IV
GAD-7: Generalised Anxiety Disorder-7 Questionnaire
HAMD: Hamilton Depression Rating Scale
MDQ: Mood Disorder Questionnaire
PH9-9: Patient Health Questionnaire-9
PI: Prinicipal Investigator
RA: Research Assistant
TAU: treatment as usual
